# Belief, Behavior, and Belonging: How Faith is Indispensable in Preventing and Recovering from Substance Abuse

**DOI:** 10.1007/s10943-019-00876-w

**Published:** 2019-07-29

**Authors:** Brian J. Grim, Melissa E. Grim

**Affiliations:** 1 Institute for Studies of Religion, Baylor University, One Bear Place #97236, Waco, TX 76798 USA; 2 Religious Freedom & Business Foundation, 1A Perry Circle, Annapolis, MD 21402 USA

**Keywords:** Substance abuse, Addiction, Faith, Valuation, Religion and spirituality

## Abstract

This study reviews the voluminous empirical evidence on faith’s contribution to preventing people from falling victim to substance abuse and helping them recover from it. We find that 73% of addiction treatment programs in the USA include a spirituality-based element, as embodied in the 12-step programs and fellowships initially popularized by Alcoholics Anonymous, the vast majority of which emphasize reliance on God or a Higher Power to stay sober. We introduce and flesh out a typology of faith-based substance abuse treatment facilities, recovery programs, and support groups. This typology provides important background as we then move on to make an economic valuation of nearly 130,000 congregation-based substance abuse recovery support programs in the USA. We find that these faith-based volunteer support groups contribute up to $316.6 billion in savings to the US economy every year at no cost to tax payers. While negative experiences with religion (e.g., clergy sex abuse and other horrendous examples) have been a contributory factor to substance abuse among some victims, given that more than 84% of scientific studies show that faith is a positive factor in addiction prevention or recovery and a risk in less than 2% of the studies reviewed, we conclude that the value of faith-oriented approaches to substance abuse prevention and recovery is indisputable. And, by extension, we also conclude that the decline in religious affiliation in the USA is not only a concern for religious organizations but constitutes *a national health concern.*

## Introduction

America is in the midst of an acute alcohol and drug addiction crisis. Life-saving medicines and psychological interventions are important components of rescue and recovery. Along with the body and the mind, the spirit is also a central part of the continuum of addiction health care. Based on our review of extensive evidence-based research on addiction that follows, it is clear that religion and spirituality—which we refer to collectively as faith—are exceptionally powerful, integral, and indispensable resources in substance abuse prevention and recovery. This body of research shows that the efficacy of faith includes not only the *behaviors* people engage in (or don’t engage in) because of their faith and the support people find in *belonging* to faith communities, but also people’s religious and spiritual *beliefs* themselves.

At the start, it is useful to provide a working definition that differentiates religion from spirituality, both of which we categorize as aspects of *faith* because the two frequently overlap, as we will show in the “Typology of Religious and Spiritual Substance Abuse Treatment Facilities, Recovery Programs, and Support Groups” section.

Spirituality is defined as an openness to God, nature or the universe where one can experience harmony with truth, feelings of love, hope and compassion, inspiration or enlightenment with a sense of meaning and purpose in life, an individual’s connection with God or the Transcendent. On the other hand, religion is viewed as the corporate expression of that connection, where one mediates their relationship to God and the community through an organized system of beliefs and practices (Burnett 2014, pp. 28–29).

## Sections of the Study

In this study, we will (1) give a brief overview of the substance abuse crisis and then (2) examine the empirical evidence illustrating faith’s contribution to preventing people from falling victim to substance abuse and helping them recover from it. After mapping the extensive literature in this field, we will (3) introduce and flesh out a typology of faith-based substance abuse treatment facilities, recovery programs, and support groups. This will provide essential background as we then (4) make an economic valuation of nearly 130,000 congregational substance abuse recovery programs in the USA.^1^ We will then (5) discuss the implications of these findings in light of the current trends of dropping religious affiliation and rising opioid addiction in the country.

### America’s Substance Abuse Crisis

**Fig. 1:**
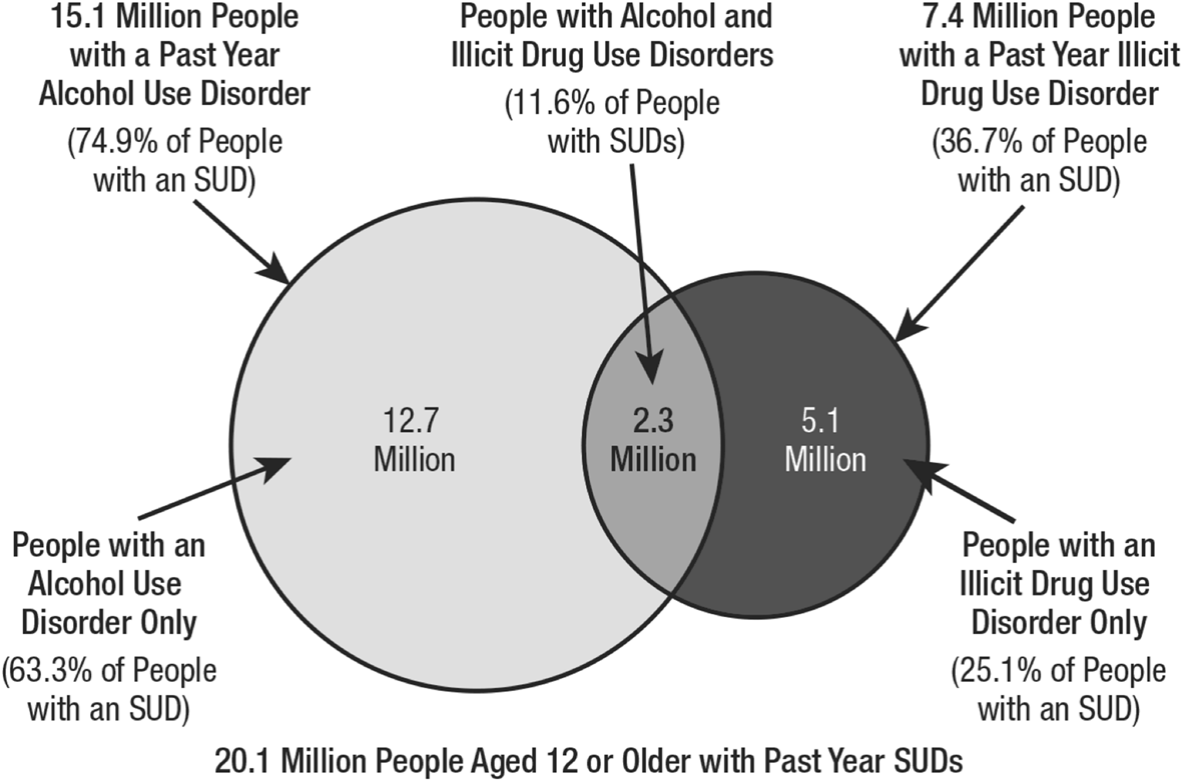
Alcohol use disorder and illicit drug use disorder in the past year among people aged 12 or older with a past year SUD: 2016. *Source*: Substance Abuse and Mental Health Services Administration (2018)

Opioid overdose deaths are only part of America’s substance abuse crisis. Nearly 1 in 10 Americans aged 12 or older (20.1 million people) have a substance use disorder (SUD), involving alcohol or illicit drugs (see Fig. 1). This includes an estimated 2.1 million people with an opioid use disorder, which includes 1.8 million people with a prescription pain reliever use disorder, according to Substance Abuse and Mental Health Services Administration (2018) of the US Department of Health and Human Services.

According to the Centers for Disease Control and Prevention (CDC) (2018a, December 19), an estimated 88,000 people (approximately 62,000 men and 26,000 women) die annually from alcohol-related causes, making alcohol the third leading preventable cause of death in the USA (after tobacco and poor diet/physical inactivity). According to the US Department of Transportation (2016a), 10,265 people died in alcohol-induced car crashes, accounting for nearly one-third (29%) of all US traffic-related deaths. The Transportation Department also estimated that an average of one alcohol-induced driving fatality occurred every 51 minutes in 2015 (National Center for Statistics and Analysis 2016).

In 2016, 63,632 drug overdose deaths occurred in the USA, a 21.5% increase from 2015. Opioids—prescription and illicit—are currently the main driver of drug overdose deaths. Opioids were involved in 42,249 overdose deaths in 2016 or 66.4% of all drug overdose deaths (Centers for Disease Control and Prevention, December 2018a). As mentioned above, the estimates indicate that drug overdose deaths continue to increase in the USA, with more than 72,000 deaths registered in 2017 (National Institute on Drug and Alcohol Abuse 2018).

Opioid overdose visits to emergency rooms increased by 30% from July 2016 to September 2017, according to the latest Centers for Disease Control and Prevention Vital Signs report (Centers for Disease Control and Prevention, March 2018b). The report notes that the Midwestern states were particularly hard hit, with a 70% increase in opioid overdose for both genders and all age-groups. The death rates also illustrate that serious thoughts of suicide are common among substance abusers, both of drugs and alcohol. In 2014, suicide was the second highest cause of death among people aged between 10 and 34 and the tenth leading cause of death in the USA overall (Piscopo et al. 2016).

### Empirical Evidence of Faith’s Contribution to Preventing Substance Abuse and Helping People Recover from It

An emphasis on the biological aspect of healing has provided us with advanced diagnostics, safe surgery, and an extended lifespan; the benefits have been extraordinary. However, these achievements often come at a cost and unnecessarily so. Disregarding the important role of the inner, spiritual aspects of healing has left developed societies with a new set of ailments, including anxiety, mood disorders, post-traumatic stress, and all sorts of addictions (Dacher 2014). However, this is changing. Writing in the American Medical Association Journal of Ethics, Robert Orr notes that “there is an increasing recognition in modern Western medicine of the importance of patient spirituality in treatment and healing” (2015, p. 414). The recognition of the significance of the spiritual aspects of healing has been growing, particularly since the 2001 mandate by the Joint Commission on Accreditation and Healthcare for the administration of a spiritual assessment by healthcare providers for patients and their families (Hodge 2006; The Joint Commission n.d.).

Hundreds of evidence-based studies demonstrate the positive impact of faith on health and well-being (e.g., Duke University n.d.; Koenig 2005, 2008, 2011, 2018; George et al. 2002; Johnson et al. 2002; Koenig et al. 2012; Rew and Wong 2006; Schoenthaler et al. 2018; VanderWeele 2017; Zemore 2008), and, as we will show in this section, nowhere is this positive impact more evident than in the recovery of people who are suffering from substance abuse. We should emphasize that the benefits of faith to health can be seen in a variety of religious contexts, including monotheistic and nontheistic faiths and beliefs. For instance, Chan et al. (2002) noted that the inner, spiritual aspects of healing are common in the Eastern philosophies of Buddhism, Taoism, and traditional Chinese medicine. Their research demonstrates significant improvements in patients when taking the body–mind–spirit integrated model of intervention. We should also note that in more than any other area of modern health care, substance abuse treatment embraces the traditional paradigm of treating body, mind, and spirit (Borkman 2008; Polcin and Borkman 2008). This is not to say that all people with addictions benefit from faith content in recovery, but many do. For example, 84% of the clients in addiction counseling expressed a desire for a greater emphasis on spirituality in treatment (Hodge 2011). Johnson and Pagano (2014) found that spiritual support and religious involvement can be an integral part of dealing with substance abuse, pertaining to both *prevention* (i.e., young adults involved in religion are less likely to become addicted to drugs) and *recovery* (i.e., addicts in spiritual programs such as the 12-step fellowships pioneered by Alcoholics Anonymous (A.A.) have a lower risk of relapse).^2^

Dr. Elinore F. McCance-Katz, Assistant Secretary of Health and Human Services for Mental Health and Substance Use, outlines three necessary steps to successfully combat and treat substance abuse in the long run: clinical care, social intervention, and social support. She highlights the strength of faith-based communities and organizations, especially in regard to social intervention and support (US Department of Health and Human Service, 2017, September 28). Government leaders recognize that the federal and state agencies are logistically unable to effectively and comprehensively confront the substance abuse epidemic on the local front where faith-based organizations work (Acker 2017; Hein 2014). By their nature, faith-based substance abuse recovery programs, particularly at the congregational level, reach beyond the addict and engage their family and community in the recovery process (White et al. 2012). As a clear indication of the US government’s ongoing recognition of the important role of faith-based communities in addressing substance abuse, the Department of Health and Human Services’ Center for Faith-Based and Neighborhood Partnerships recently published an *Opioid Epidemic Practical Toolkit: Helping Faith and Community Leaders Bring Hope and Healing to Our Communities* (US Department of Health and Human Services, 2018a, August 3).

We will now specifically look at the evidence-based research on (a) how faith *generally* relates to substance abuse, (b) how faith relates to *youth* and substance abuse, and (c) how faith relates to *adults* and substance abuse; we will also provide an overview of the available evidence-based studies on the effectiveness of faith-based substance abuse recovery support programs.

#### Faith’s Relationship with Substance Abuse in General

Evidence-based studies point to the instrumental contribution of faith to substance abuse prevention and recovery. A large majority of cases show that religious and spiritual beliefs and practices lead to lower levels of substance abuse, including reduced likelihood of using various drugs, in the course of a lifetime (Degenhardt et al. 2010; Herman-Stahl et al. 2007; Moscati and Mezuk 2014; Palamar et al. 2012). For instance, a study by Lyons et al. (2010) found that up to 82% of clients who experienced a spiritual awakening during substance abuse treatment and recovery were completely abstinent at a 1-year follow-up compared with 55% of non-spiritually awakened clients.

**Fig. 2:**
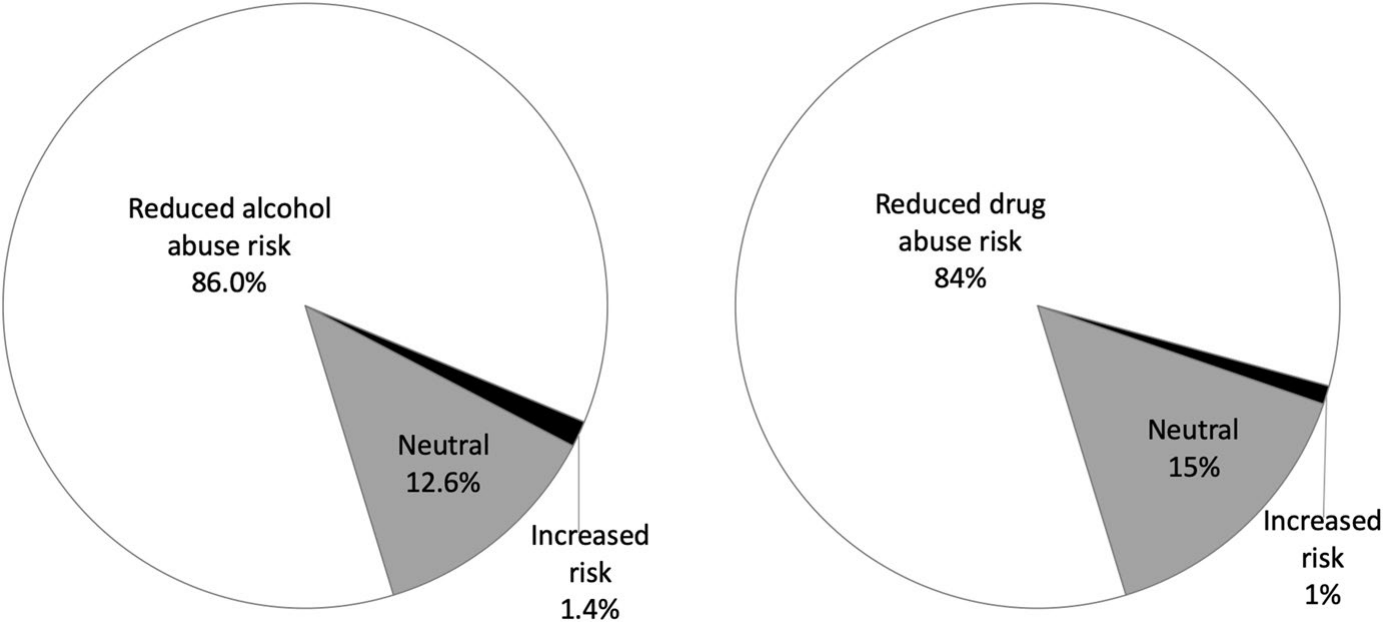
Findings from studies on the impact of faith on substance abuse. *Data Source*: Koenig et al. (2012); number of studies reviewed: alcohol (278), drugs (185)

Koenig et al. (2012) identified at least 278 quantitative studies that attended to the relationship between alcohol abuse and faith prior to 2010. Of these, 86% found that faith reduced the risks associated with alcohol use, abuse, or dependence; only four studies (1.4%) found that faith contributed to alcohol use, abuse, or dependence, with the rest being neutral (see Fig. 2). It is possible that the findings reported on the positive role of faith were arrived at through less rigorous methods. However, to test this, the authors looked separately at only 145 research of highest quality among the 278 evidence-based studies. Among these, 131 (90%) found that faith reduced the risks of alcohol use, abuse, or dependence, while only one found that faith contributed to alcohol use, abuse, or dependence.

Koenig and colleagues (op. cit.) also reviewed the studies that examined the relationship between faith and drug use, abuse, or dependence, and as Fig. 2 shows, they obtained similar results. Of the 185 studies identified, 84% found that faith reduced the risks of drug abuse and only two (1.4%) found that faith contributed to drug abuse.

These findings echo those of Rew and Wong (2006) who found that, among 43 studies, most (84%) showed that religiosity, i.e., the intensity of religious involvement and practice, and/or spirituality had positive effects on health attitudes and behaviors. The results are similar to an earlier review by Moody-Smithson (2001) of more than 100 studies prepared for the Center for Substance Abuse Treatment (CSAT), which found that 90% of the studies reported that substance abuse was less common among more religious people. The inclusion of faith-based elements in otherwise secular programs has also been shown to be effective. For example, Nemes et al. (1999) studied two 12-month substance abuse treatment programs and found that the clients who completed all the components of treatment, including faith-based elements, reported less substance use at subsequent follow-ups.

Some of the studies with neutral or negative findings include those with mixed results. For instance, a study by Yeterian et al. (2018) found that among adolescents being seen in an outpatient SUD program, higher baseline spirituality predicted a lower likelihood of heavy drinking at follow-up, even more so than religiosity. However, higher levels of religion and spirituality at the baseline were related to increased marijuana use at the 6-month follow-up, as participants reported that they felt more spiritually connected when they were high on marijuana. Another recent mixed-results study of 1565 young black homosexual men in Houston and Dallas showed that participation in spiritual and religious activities is an important source of resilience, albeit a risk for these men (Carrico et al. 2017). On the one hand, the odds of substance use diminished when the men had higher levels of “spiritual coping” (i.e., the ability to tap into spiritual support and look for meaning in a traumatic situation). On the other hand, more engagement in spiritual and religious activities was also found to be associated with greater odds of substance use because of the huge stigma associated with being black and gay.

#### Faith’s Relationship with Substance Abuse Among the Youth

Evidence-based studies have found that youths who are spiritually active, participate in a faith community, and invest in a prayerful relationship with their God are less likely to use or abuse drugs and alcohol and engage in related criminal activity (Johnson et al. 2015, 2016a, b; Lee et al. 2014, 2017; Post et al. 2015, 2016). A seminal 2-year study by The National Center on Addiction and Substance Abuse (2001) at Columbia University, directed by Joseph A. Califano Jr., the former US Secretary of Health, Education, and Welfare in the Clinton administration, found that the teens who did not consider religious beliefs important were almost three times more likely to smoke, five times more likely to binge on alcohol, and almost eight times more likely to use marijuana compared with the teens who strongly appreciated the significance of religion in their daily lives. The study also found that, compared with the teens who attended religious services at least weekly, the teens who never attended services were twice more likely to drink, over twice more likely to smoke, over three times more likely to use marijuana or binge on alcohol, and four times more likely to use illicit drugs.

A host of studies show that faith among adolescents and young adults can act as a powerful deterrent against drug and alcohol abuse, even when controlling for other contributory factors (e.g., depression). One of the largest studies on drug and alcohol abuse among American youth aged 12–17 analyzed data from the National Survey on Drug Use and Health (Ford and Hill 2012); the study found that higher degrees of religiosity reported, including religious attendance, involvement, and reliance on religious beliefs in decision making, were associated with several benefits, such as limited depression and negative attitudes toward substance abuse. After controlling for depression, religiosity was still found to be associated with less cigarette smoking, heavy drinking, and prescription and illicit drug abuse. Adolescents who frequently attend religious services, who are involved in faith-based activities, and who place a high value on spirituality exhibit greater resilience when facing the stressors that can lead to the formative use of drugs and alcohol as a coping mechanism. A web-based survey of 5217 students in grades 6–8 at parochial private schools in the Baltimore Area, conducted at Johns Hopkins School of Public Health and Center for the Prevention of Youth Violence (Debnam et al. 2016), examined the associations between stress, spirituality, and substance abuse. The research found that, while stress was a predictor of substance abuse, it even had a stronger correlation with substance abuse for students who reported lower spiritual beliefs. Similarly, a study of Native American youths in grades 7–8 revealed that involvement in religious practice and native culture helped them better integrate into society and protect themselves against substance abuse (Kulis et al. 2012).

Investigators at the University of Virginia, Johns Hopkins School of Public Health, and Cedars-Sinai Medical Center in Los Angeles (Debnam et al. 2018) examined the moderating effect of spirituality on the relationship between psychological stress and substance use among 27,874 high school students from the state of Maryland. They discovered that higher spirituality was related to lower substance abuse in both males and females and further moderated the effect of stress that would have otherwise culminated in substance abuse by males. Moreover, youths and teens who have been religiously active and who have made prayer and belief in God an integral part of their lives have better coping mechanisms when attending drug rehabilitation programs and better outcomes after the programs end. For instance, a controlled study of substance-dependent youths revealed that those who had been assessed at the baseline with preexisting greater lifetime religious involvement were more likely at the end of the treatment to be regularly engaged in abuse recovery activities and behaviors, predicting greater recovery outcomes (Kelly et al. 2011).

These are not isolated findings. There is overwhelming evidence that religious involvement and/or religiosity are associated with reduced risk of substance use among adolescents (Bahr and Hoffmann 2008; Bartkowski and Xu 2007; The National Center on Addiction and Substance Abuse 2003; Metzger et al. 2011; Steinman and Zimmerman 2004; Wallace et al. 2007). The teens who attend religious services weekly are less likely to smoke, drink, use marijuana or other illicit drugs (e.g., LSD, cocaine, and heroin) than the teens who attend religious services less frequently (Brown et al. 2001; The National Center on Addiction and Substance Abuse 2010; Longest and Vaisey 2008; Steinman et al. 2006; Wills et al. 2003). Further, religious practice among teens discourages them from taking highly dangerous drugs (Adlaf and Smart 1985; Thompson 1994). In their study, Chen and VanderWeele (2018) found that people who attended religious services at least weekly in childhood and adolescence were 33% less likely to use illegal drugs. Adolescents also benefit from their mothers’ higher levels of religious practice, controlling for factors that also influence the level of drinking (e.g., the adolescents’ peer associations) (Foshee and Hollinger 1996). Higher teenage religiosity was also related to other factors related to a decrease in drug use, such as good family relations, high academic performance in school, having anti-drug attitudes, and socializing with friends who do not take drugs (Johnson 2002). Moreover, teens themselves tend to cite their peers’ religious and spiritual inclinations as reasons that discourage their peers from drinking and taking drugs (The National Center on Addiction and Substance Abuse 2011).

#### Faith’s Relationship with Substance Abuse Among Adults

The 2001 National Center on Addiction and Substance Abuse study found that the adults who do not consider religious beliefs important are more than three times more likely to binge on drinks and almost four times more likely to take illicit drugs. The study also found that, compared to those who attend religious services at least every week, the adults who never attend religious services are more than five times more likely to take illicit drugs and almost seven times more likely to binge on drinks. The study found that people “with strong religious or spiritual beliefs are healthier, heal faster and live longer than those without them” and that “religion and spirituality can play a powerful role in the prevention and treatment of substance abuse and in the maintenance of sobriety” (The National Center on Addiction and Substance Abuse 2001, p. ii).

Faith protects both women and men against substance abuse. A study of over 11,000 women, aged 18 and older, found significant reductions in alcohol and drug use by more religiously active women, including lesbian and bisexual women (Drabble et al. 2016). Acheampong et al. (2016) showed that women and men who use prescription opioids and who are actively religious and spiritual are less likely to engage in simultaneous polysubstance use (SPU). The protective feature of religious engagement against alcohol abuse can have a lasting impact. For example, Koenig and Vaillant (2009) found that frequent religious attendance at midlife (ages 45–47) was protective against alcoholism and predicted a significant increase in subjective well-being by the age of 70, independent of other predictors and baseline well-being.

Research also indicates that religious engagement can be especially useful for minority populations in the USA. In their analysis of the cross-sectional data from a nationally representative sample of 868 Latinos of Mexican origin from the National Latino and Asian American Study (NLAAS), Moreno and Cardemil (2018) found that religious attendance was linked to lower lifetime prevalence of depressive disorder, anxiety disorder, and SUD. A qualitative study by Cheney et al. (2013) delved into some of the religious and spiritual dimensions of reducing and abandoning cocaine use among African–Americans in rural and urban areas of Arkansas. Their analysis suggested four ways in which religion could have an impact. First, the participants *situated substance use in religious and spiritual frameworks.* Second, *participation in organized religious activities helped* many of the participants cut down on or briefly stop cocaine use at some point in their substance use history. These activities ranged from attending church and Bible studies to singing in the choir, which they identified as a steadying force that helped reduce cocaine use. Third, the *participants cited their personal relationship with God* as a factor in reducing cocaine use and placing them on the road to recovery. And fourth, many *participants expected God to step in.*

Research shows that a person’s effective use of the spiritual resources from their faith tradition—positive religious coping (PRC)—contributes to better substance abuse recovery outcomes. Religious coping may range from prayer to convictions of religious faith and belief itself (Elmholdt et al. 2017; Schjødt et al. 2008, 2009; Jegindø et al. 2012; Yu et al. 2016). People who use PRC tend to seek spiritual support and meaning when inflicted by traumatic events; by contrast, people who resort to negative religious coping (NRC) can have a hard time recovering, as they experience spiritual complications and express doubt about the issues of God and faith. For instance, Medlock et al. (2017) found that PRC had a positive correlation with a patient’s reduced cravings and increased productive participation in 12-step meetings, whereas patients who relied on NRC suffered withdrawal symptoms more acutely and benefited less from the 12-step meetings.

PRC has been shown to help maintain sobriety during the postdrug rehabilitation period. Martin et al. (2015) followed participants in alcohol outpatient treatment from 2 weeks until 6 months after enrollment; they found that those participants who relied on religion to help them cope were less likely to drink heavily and had fewer drinks per day than those who believed in no religion or resorted to NRC. PRC has also been found effective when dealing with opioid dependence—an addiction with high rates of relapse. While there are pharmacotherapies, such as methadone and buprenorphine, which are effective in reducing relapse, they are not effective enough in isolation for treating the whole person. Puffer et al. (2012) found that increased PRC was associated with less frequent opioid use and more frequent 12-step participation. They also found that patients who were able to decrease NRC were less prone to relapse.

International studies provide corroborative support for US findings, linking religious participation and a personal prayerful connection with God (or spirituality) to fewer addictive behaviors (e.g., Szaflarski 2001; Gomes et al. 2013; Haug et al. 2014).

#### Effectiveness of Selected Faith-oriented Substance Abuse Recovery Support Programs

*Religion*-*based* substance abuse recovery programs include those that are carried out by such groups as the Salvation Army and Teen Challenge (Adult and Teen Challenge USA 2018), and *spirituality*-*based* programs include those carried out by such groups as A.A. and Narcotics Anonymous (N.A.). A survey of the Salvation Army’s Harbor Light Center in Washington D.C. (Wolf-Branigin and Duke 2007) found that participants who chose engagement in spiritual activities improved their chance of successfully completing their treatment program.

In 2017, Teen Challenge USA helped, on average, 5826 individuals in their US residential programs each day (Teen Challenge 2018, p. 1). A 7-year study on Teen Challenge’s effectiveness found that, in contrast to those who had dropped out of the program, the program’s graduates had significantly managed to alter their behavior (Bicknese 1999). A Teen Challenge survey (Owen et al. 2007) revealed that the top two factors in maintaining sobriety after rehabilitation were staying connected to God (58%) and family (34%). At the time of the follow-up contact, an average of 2.7 years after graduation, 45% of the participants stated they had not had a single relapse; 92% of the participants said that their drug use was “a lot less” and 83% said that their drug use was “a lot less” than before Teen Challenge. The results of this research are further supported by a follow-up Teen Challenge survey (Hardeman et al. 2011) that found that the top three factors in maintaining sobriety after rehabilitation were “staying connected to God” (62%), “family” (36%), and “hanging out with positive people” (22%). The graduates in general found the faith-based elements most useful for helping them recover.

A.A. is not only the most widely used spirituality-based support/mutual aid group for people recovering from alcoholism, but also provides inspiration for a multitude of other addiction recovery support groups (BBC Magazine 2015; Laudet 2008). Although some have questioned the usefulness of A.A. (Anderson 2015; Cunha 2015; Dodes and Dodes 2014; Ferri et al. 2006), the effectiveness of its approach has solidly been established in an edited volume by Galanter and Kaskutas (2008) for the American Society of Addiction Medicine and the Research Society on Alcoholism. The volume provides an overwhelming body of theoretically informed and evidence-based empirical research, demonstrating the effectiveness of A.A. and its original spirituality-based 12-step approach as well as spirituality’s general role in addiction recovery. The volume also shows that A.A. has significantly informed and influenced how alcoholism is professionally treated today (Slaymaker and Sheehan 2008). There are numerous other empirical studies on A.A.’s effectiveness. First, based on a 13-item A.A. Involvement Questionnaire of the extent of participants’ involvement in A.A. (Tonigan et al. 1996), the level of participation in A.A. is a determinant of a patient’s treatment outcome (Montgomery et al. 1995). Additionally, for adults who struggle with addiction, The National Center on Addiction and Substance Abuse (2001) found that the individuals who had received both professional treatment and attended spirituality-based support programs like A.A. or N.A. were far more likely to stay sober than if they had received professional treatment alone.

Kaskutas et al. (2003) examined the role of religiosity in A.A. involvement and long-term sobriety in a representative sample of 587 men and women interviewed upon entering treatment and re-interviewed one and 3 years later. Those who reported a spiritual awakening at Year 3 had had the highest chance of continued sobriety for the past year, a state that would not be equally extended to mere religious self-definition. The study also found that an increase in A.A. activities, besides just attending A.A. meetings (e.g., sponsorship), between the baseline and the first-year follow-up was also associated with greater likelihood of sobriety. For instance, the supportive, helping behaviors encouraged by A.A. were seen by members as an expression of spirituality in the recovery context (Zemore et al. 2004). White and Kurtz (2008) observed that a defining moment in the history of A.A. was connected to the realization of psychoanalyst Carl Young who, after providing the best treatment that psychiatry and medicine could offer, still saw a patient he treated relapse (Galanter and Kaskutas 2008). He then observed that patients maintained sobriety successfully through religious and spiritual experiences. Research indicates that atheists and agnostics benefit from the support for a sober lifestyle in A.A. groups as equally as religious people (Tonigan et al. 2002; Borkman 2008). Hsu et al. (2008) also showed how the mindfulness and meditation inherent in the 12-step approaches are natural features of Buddhism and Buddhist approaches to addiction recovery. Built into A.A. is the idea that all members are recovering from alcoholism and none has moral superiority (White and Kurtz 2008), with all unified by a single membership criterion—the desire to stop drinking.

Since volunteering and helping others are associated with positive health outcomes (Yeung et al. 2017), substance abuse recovery programs dependent on volunteering, such as those offered in A.A. and congregation-based groups, have a built-in advantage for success. More specifically, volunteering and helping others is found to be instrumental in addiction recovery (Lee et al. 2016; Johnson et al. 2016a, b; Pagano et al. 2015; Post et al. 2015, 2016). From the perspective of social identity theory (Dingle et al. 2015), recovery is aided, and perhaps necessitated, by the presence of a consistent reference group of individuals who can help patients reconstruct their new identity as “nondrinking alcoholics,” that is, someone who is prone to abuse alcohol but decidedly no longer drinks (Borkman 2008). In fact, those overcoming a troubled past can be a limitless source of help and inspiration for those still struggling (Zemore and Pagano 2008).

It’s important to note that being vaguely spiritual is not itself indicative of behavior change (Jang and Franzen 2013): spirituality needs concrete beliefs (religious or religion-like), behaviors, and/or belongings in order to change outcomes. A.A. has all three elements in the form of a set of beliefs summarized in its Big Book, behaviors expected of members such as cessation of drinking, and belonging, such as all encouraged to have a home group (Alcoholics Anonymous n.d.).

### Typology of Religious and Spiritual Substance Abuse Treatment Facilities, Recovery Programs, and Support Groups

We will now provide an overview of religious and spiritual substance abuse treatment in the USA, which is necessary because, in the “Valuation” section, we use data from A.A. as a proxy for congregationally provided recovery support groups, which can be religious, spiritual, or both.

**Fig. 3:**
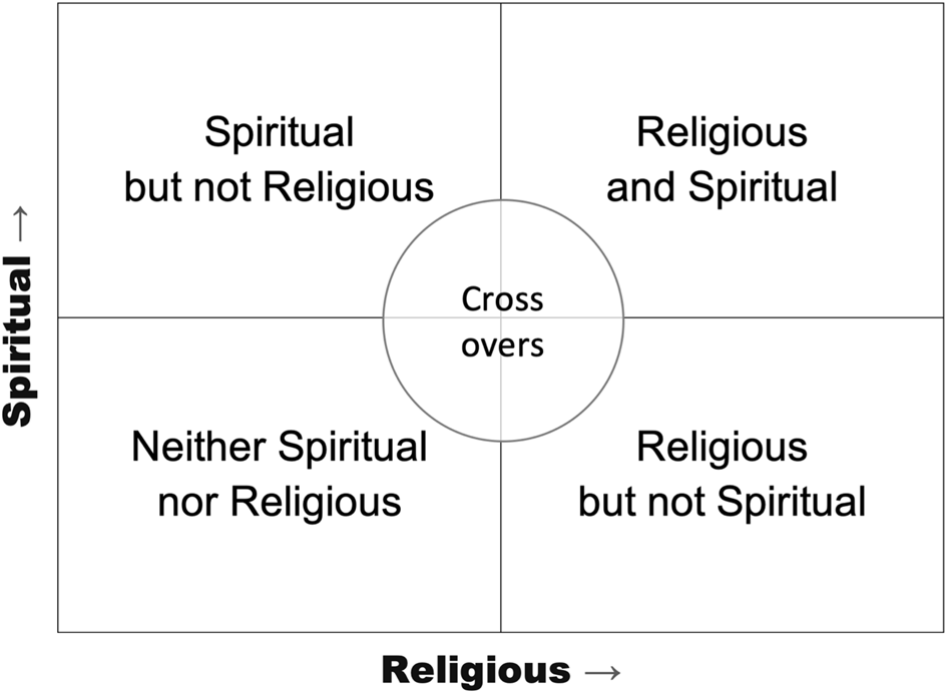
Typology of faith-oriented substance abuse treatment facilities, recovery programs, and support groups

The Department of Health and Human Services’ database of more than 14,500 specialized facilities for persons suffering from SUD provides barely any searchable information on whether the providers incorporate a religious element or whether the provider is a faith-based organization (Substance Abuse and Mental Health Services Administration n.d.c). In fact, there is no central directory or coordinating body that reports on or tracks faith-based initiatives and their effectiveness. Therefore, to better understand the role of spirituality, religion, and faith-based interventions in substance abuse treatment and recovery, we propose a typology for treatment and recovery programs and support groups along the vertical axis of spirituality and the horizontal axis of religiosity (see Fig. 3). This typology aids in understanding how religion and spirituality are related phenomena and not mutually exclusive (e.g., Hodge 2011; Koenig et al. 2001; Richards et al. 2009). Spirituality is also comfortable to many who have a secular orientation. For instance, A.A. Agnostics of the San Francisco Bay Area (2018) considers its branching communities as “spiritual” programs. The definition provided in the “Introduction” section illustrates the overlaps between religion and spirituality.

Furthermore, religion in its own right can serve as a repository and center of spirituality, such as Ignatian spirituality nurtured by the Society of Jesus (the Jesuits n.d.), mysticism as practiced in Sufi Islam (Cook 2015), or the exercise of Buddhist meditation techniques (Amihai and Kozhevnikov 2015). With this in mind, addiction treatment and recovery programs can range from the ones that are (a) spiritual but not religious, (b) religious and spiritual, (c) religious but not spiritual, or (d) neither religious nor spiritual. This section will describe and provide examples of each. It is important to note that not every scenario neatly fits each quartile; some may cross over and overlap others, hence “crossovers” in the center of the taxonomy.

#### Spiritual but Not Religious Substance Abuse Recovery (Ex. 12-Step Programs)

The vast majority of state-of-the-art substance abuse treatment and recovery programs in the USA include a key component that is spiritual but not necessarily religious (i.e., the 12-step recovery assistance program that A.A. and N.A. have developed and popularized). Our analysis of the Substance Abuse and Mental Health Services Administration (SAMHSA) database shows that 73% of the behavioral health substance abuse treatment services in the USA include a 12-step program or option (see Table 1). Although A.A. and N.A. are neither faith-based nor religious organizations, seven of their 12 steps explicitly mention God, a Higher Power, or spirituality. In fact, A.A. has clear roots in Protestant and Catholic Christian thought and practice (Burnett 2014; Chesnut 2014) and is predicated on the need for a Higher Power to help alcoholics become and remain sober. While this Higher Power is God for many members of the 12-step programs and fellowships, atheists and other nontheistic A.A. participants may define their Higher Power as the collective strength and support provided in their group meetings.

**Table 1:** 12-step programs among substance abuse programs (among those in the SAMHSA database, May 2018). *Source*: Substance Abuse and Mental Health Services Administration (SAMHSA n.d.c.)

Populations served	12-step program reported?				Total
	Yes		No		
	*N*	%	*N*	%	
Overall	9046	73	3272	27	12,318
Persons with HIV or AIDS	1798	80	437	20	2235
Military families	1153	79	298	21	1451
Veterans	1855	79	480	21	2335
Persons who have experienced sexual abuse	2485	79	667	21	3152
Seniors or older adults	1943	79	523	21	2466
Lesbian, gay, bisexual, or transgender (LGBT) clients	1853	79	504	21	2357
Adult men	4493	79	1230	21	5723
Active duty military	986	78	272	22	1258
Adult women	4723	78	1315	22	6038
Persons who have experienced trauma	3703	78	1038	22	4741
Persons who experienced intimate partner/domestic violence	2460	78	709	22	3169
Transitional age young adults	2818	78	813	22	3631
Pregnant/postpartum women	2200	78	637	22	2837
Persons with co-occurring mental and substance abuse disorders	4746	77	1390	23	6136
Clients referred from the court/judicial system	3361	76	1033	24	4394
Young adults	6558	75	2156	25	8714
Adults	8788	74	3032	26	11,820
Female	8399	73	3079	27	11,478
Male	8436	73	3099	27	11,535
Adolescents	2280	70	967	30	3247
Children/adolescents	3496	70	1499	30	4995

As shown in Table 1, twelve-step recovery programs are common across all substance abuse treatment programs, including programs serving vulnerable populations, such as persons who have experienced sexual abuse or persons living with HIV/AIDS.

As our typology suggests, A.A. and N.A. groups can range from those with no religious content, such as A.A. Agnostics of the San Francisco Bay Area, mentioned above, to those that have overt religious content such as closing with the Lord’s Prayer. The only requirement for A.A. membership is a desire to stop drinking. All A.A. and N.A. groups are self-supporting and do not accept contributions from non-A.A. members, and, most importantly, because they are locally administered, each group may have a slightly different character.

While A.A. and N.A. groups are not religious organizations, they frequently meet in spaces provided by local congregations at a low or no cost.^3^ In effect, many of the nearly 130,000 US congregations that have alcohol and drug abuse recovery groups (Grim and Grim 2016) either host A.A. or N.A. meetings or offer their own version of the 12 steps. When a church hosts an A.A. meeting, even though it has no programmatic oversight, churches still consider this as something the church is facilitating. This is an example of the same activity crossing over from one type of program to another, as shown in the typology above (Fig. 3). For instance, our review of the A.A. meetings in Nashville, Tennessee, suggests that, of the 64 different facilities hosting A.A. groups, 51 (80%) are churches or other religious properties (A.A. Nashville n.d.). This is not surprising, given that the majority of people in the Nashville Area are members of some religious denominations (Grammich et al. 2012); less expected, though, is the fact that in Seattle, Washington, where the majority of people are religiously unaffiliated, by our analysis, 29 (54%) of the 54 facilities hosting A.A. groups are churches or other religious properties (A.A. Seattle n.d.). The Appendix Table 5 provides a detailed example of the support provided by local congregations to Appendix Table 5 in Annapolis, Maryland, where the authors live. The 160 weekly meetings outlined in the Appendix’s Table 5 represent more than half (57%) of the 281 A.A. meetings held in the Annapolis Area each week (Annapolis Area Intergroup 2017). It is also not unusual for some congregational properties to serve as a hub for A.A. in a region. In historic downtown Annapolis, one of the buildings on the campus of the First Presbyterian Church, known as the Red House, serves as a permanent place for A.A. meetings in the area and holds the offices of the area’s A.A. Intergroup (akin to a regional organizing committee). Although these A.A. meetings occur inside the church buildings, the programs offered are spiritual and not religious; in other words, they are not based on the religion or religious oversight of the churches hosting the groups.

#### Religious and Spiritual Substance Abuse Recovery (Ex. Religion-Based 12-Step Programs)

A number of religion-based 12-step programs have taken the spiritual elements of A.A. and made them overtly religious. For example, Monty Burks, the Director of Faith-Based Initiatives at the Tennessee Department of Mental Health and Substance Abuse Services (TDMHSAS), noted in our interview with him that across Tennessee there is a widespread use of Celebrate Recovery, a 12-step recovery assistance program adapted from A.A. and N.A. Celebrate Recovery, developed at Saddleback Church in southern California, offers an explicitly evangelical Christian version of the 12-step program. To date, over 5 million individuals are reported to have completed a Celebrate Recovery Step Study (Celebrate Recovery n.d.).

Tennessee also offers an example of a close working relationship between faith communities and a state government, aimed at addressing substance abuse. TDMHSAS’ Office of Faith-Based Initiatives engages communities of faith across the state and certifies them as being qualified to meet the recovery needs of the people in their pews and in their area (TDMHSAS n.d.). As of 2017, about 250 recovery churches or congregations have been certified by TDMHSAS in Tennessee and about 50 others are in the process of becoming certified (Morris 2017). Across Tennessee’s 95 counties, a church or faith-based organization is the only civic institution common in all counties: “So, we figure that’s a perfect vehicle to drive information into the community and teach people that you don’t have to be an addiction counselor to help somebody who’s an addict,” said Monty Burks (in Vance 2016, paragraph 21).

Another religious adaptation of the A.A.’s 12-step recovery program is the Addiction Recovery Program of The Church of Jesus Christ Latter-day Saints. Unlike A.A. or N.A. programs, the Latter-day Saint’s program invites people who are struggling with all forms of addiction (e.g., drugs, alcohol, pornography, gambling, eating disorders, etc.) to attend the same meeting together. The program has adapted the 12 steps of A.A. into the framework of the doctrines, principles, and beliefs of the Church (The Church of Jesus Christ Latter-day Saints 2018).

Christian groups are not the only faith traditions to adopt and adapt the A.A.’s 12-step approach. Beit T’Shuvah, a residential 140-bed Jewish addiction treatment center and congregation, incorporates Jewish spirituality into its 12-step program. Millati Islami World Services, founded in Baltimore, Maryland, is a 12-step recovery program based upon Islamic principles (Ali 2014; Millati Islami World Services n.d.). Millati Islami reports that its modified 12 steps and traditions, which incorporate Islamic principles, are of great benefit to Muslims in recovery.

A.A.’s spirituality-infused 12-step recovery programs have inspired numerous nontheistic programs as well. Native American communities have used modifications of the steps to address the historical trauma they have experienced that contributes to their increased rates of depression, drug use, and addiction (see Acker 2017). The Wellbriety Movement (n.d.), for example, is a 12-step program that incorporates Native American cultures and spirituality (Sacred Connections n.d.). Another nontheistic adaptation of A.A. approach are *Mindfulness and 12 Steps,* weekly meetings that explore the basic teachings of the Buddhist practice of mindfulness and the participants’ own reflections in the twelve steps (Buddhist Recovery Network n.d.).

#### Religious but Not Spiritual Substance Abuse Treatment (Ex. Faith-Based Hospitals)

CHI St. Gabriel’s Health Opioid Program in rural Minnesota is a classic example of a religious but not spiritual substance abuse treatment and recovery program. St. Gabriel’s is recognized nationally for leading a faith-based charge against opioid abuse (American Hospital Association n.d.; Oosten 2018; Rioux 2018). The hospital does not advertise its Catholic identity in its name, although CHI is an acronym for Catholic Health Initiatives. And when hospital staff were interviewed for this study, the program was not described in spiritual terms but in medical and community terms, as a manifestation of its overall Catholic approach. The Catholic identity of the hospital is summarized in its mission statement: *“*The mission of CHI St. Gabriel’s Health is to nurture the healing ministry of the Church …. Fidelity to the Gospel urges us to emphasize human dignity and social justice as we create healthier communities” (CHI St. Gabriel’s Health n.d.).

Being “religious but not spiritual” is common to many religion-based institutions receiving government funding. Another example is the *For My Baby and Me* program, launched by Trenton Catholic Charities in December 2017 with a $1 million New Jersey Department of Health grant (Diocese of Trenton 2018). In our interviews, the program staff pointed to the type of population that is served as a direct result of their Catholic mission to serve the most vulnerable and neglected. *For My Baby and Me* seeks to meet critical needs pregnant, addicted women because very few recovery programs accept pregnant women, who require complex, specialized care (Capital Health n.d.). Further, the Catholic concern for life from conception to natural death also led the team to focus on this particular population because by caring for the pregnant mother, two and not just one life is saved.

#### Substance Abuse Recovery Programs with Little or No Religious or Spiritual Content

While we are primarily focused on bringing to light religious and spiritual contributions to substance abuse recovery, we also believe that programs without these elements are valuable and helpful to many people, including religious people. The Secular Organization for Sobriety (S.O.S.) is an alternative to the 12-step model of recovery but “welcomes the attendance of religious, as well as nonreligious persons” (Secular Organization for Sobriety n.d., paragraph 3). LifeRing, also a secular program, reports that about 40% of their participants attend a house of worship, according to a 2005 survey (LifeRing n.d.). In general, these programs, including SMART Recovery, focus on an individual’s ability to take charge of their recovery, as suggested by the term “SMART” in SMART Recovery, which is an acronym that stands for “Self-Management and Recovery Training.” As with LifeRing and S.O.S., participants can be religious; however, “if you do not believe in a religion or spirituality, that’s fine” (SMART Recovery n.d., paragraph 1).

### Valuation

As we have just demonstrated, not only does faith offer personal and social resources helping people avoid and/or recover from substance abuse, its impact is often made manifest at the local congregational level, as places of worship host spiritual or religious 12-step type fellowship meetings. We will now build on and extend the work of Grim and Grim (2016), who produced the first economic valuation of the contribution of religion to American society at the national level. The study put the annual contribution in dollar terms, with a mid-range estimate of nearly $1.2 trillion. This includes the fair market value of community services provided by religious organizations such as an estimated 129,680 congregational substance abuse recovery programs (2016, p. 17). The study did not, however, conduct a valuation of each type of community service, but rather used an algorithm based on in-depth studies of individual congregations by Cnaan et al. (1999, 2006, 2013) and Cnaan (2015). Johnson (2016) argued, however, that while the study was valuable and groundbreaking, it *undervalued* the economic impact of religion.

#### Valuation of Congregation-Based Substance Abuse Recovery Mutual Support Programs

We will now make a valuation of these congregation-based programs drawing on the same methodology used by the Council of Economic Advisors (2017) to put a dollar value on America’s opioid crisis.

#### White House Valuation of Opioid Crisis Based on Value of a Statistical Life (VSL)

Within the Executive Office of the President, the Council of Economic Advisers (CEA) is charged with offering the President objective economic advice on the formulation of both domestic and international economic policy using the best data available. The CEA chairpersons require Senate confirmation. In November 2017, the CEA issued a report (Council of Economic Advisers 2017) offering a new valuation of the adverse impact of the opioid crisis on the American economy, titled “The Underestimated Cost of the Opioid Crisis.” The brief 14-page report changed the national discussion on the crisis by putting a $504 billion value on the human cost of substance abuse. While there is no perfect methodology for estimating the cost of a lost or ruined human life, over the years researchers have reached a consensus that economic valuations of a fatality, i.e., the value of a statistical life (i.e., VSL) is in millions of dollars (Viscusi 2013).^4^ VSL is used by various government agencies to estimate the economic cost–benefit value of certain risk-reduction policies, such as the economic value of lowering speed limits to reduce traffic fatalities or building a levee to prevent catastrophic flooding or, in this case, spending money on substance abuse prevention to save lives.^5^ The 2017 CEA report reviewed research on the range of empirical estimates of the VSL used by the federal government regulatory and health agencies in order to estimate the economic cost of the opioid crisis (Robinson and Hammitt 2016; Viscusi 2015; Viscusi and Aldy 2003; Viscusi and Masterman 2017). The CEA report (2017, p. 4) identified three federal agencies that have issued formal guidance on VSL to inform their rulemaking and regulatory decision making: the US Department of Transportation (DOT), the US Environmental Protection Agency (EPA), and the US Department of Health and Human Services (HHS).^6^

In the end, the White House presented cost estimates under three alternative VSL assumptions: low ($5.4 million), middle ($9.6 million), and high ($13.4 million), based on the US DOT and similar to those used by HHS. Thus, their low fatality cost estimate of $221.6 billion is the product of the adjusted number of fatalities (i.e., 41,033) and the VSL assumption of $5.4 million. Their fatality cost estimates thus range from a low of $221.6 billion to a high of $549.8 billion, which is the product of fatalities and the high estimate. Their estimates also take into account that opioid fatalities are more common among younger age-groups, as also shown in the same table under the age-dependent VSL assumption. Finally, the CEA estimate includes non-fatality costs in addition to the cost of fatalities each year. They estimated those costs by using the estimates of Florence et al. (2016) to calculate a measure of per-person costs of opioid misuse among those who did not die within the year and then multiplying that per-person cost by the number of individuals with an opioid use disorder in 2015. Florence et al. (2016) estimates of increased costs due to prescription opioid misuse were $58.0 billion (according to the 2015 value of dollar), broken down as follows: $29.4 billion: increased health care and substance abuse treatment costs; $7.8 billion: increased criminal justice costs; and $20.8 billion: reduced productivity among those who do not die of an overdose.

The CEA took this non-fatal total cost of $58.0 billion and divided it by the 1.9 million individuals who had a prescription opioid disorder in 2013 (the reference year of Florence et al. (2016) study), resulting in an average cost of approximately $30,000 per person. The CEA applied that average cost to the 2.4 million people with opioid disorders in 2015, resulting in a total cost of $72.3 billion for non-fatal costs (the CEA also included heroin disorders as well as prescription opioid misuse).

#### Valuation of Congregation-Based Substance Abuse Recovery Support Programs

Using the White House CEA’s methodology as a blueprint, we can estimate an economic valuation of congregation-based abuse recovery support programs’ contribution to American society and its economy. Detailed data are not available for the nearly 130,000 congregational substance abuse recovery groups. However, data are available for A.A., which has been conducting surveys of their members every 3 to 4 years since 1968 (Alcoholics Anonymous 1970). A.A. conducts these surveys to keep members informed of the current membership trends. As a proxy, the A.A.’s surveys, in combination with other data summarized in Table 2, are particularly useful for making valuation of religious and spiritual substance abuse recovery programs, mainly because many A.A. groups meet in churches and other faith congregations (see the “Spiritual but Not Religious Substance Abuse Recovery (Ex. 12-Step Programs)” section).^7^

**Table 2:** Data used in proxy valuation of religious and spiritual substance abuse recovery programs held in congregations

Data	Source
Length of sobriety	Alcoholics Anonymous (2014)
Risk of relapse over time	Dennis et al. (2007)
Age structure of A.A. members	Alcoholics Anonymous (2014)
Mortality rates	National Vital Statistics Reports (Xu et al. 2018)
Relative mortality risk of people with alcohol use disorder	Laramée et al. (2015)
Total membership	Alcoholics Anonymous (2018a)
Numbers of groups	Alcoholics Anonymous (2018a)
VSL	CEA (2017)
Non-fatality costs of addiction	CEA (2017)
Number of religious and spiritual substance abuse recovery programs held in congregations	Grim and Grim (2016)

We will now go through a series of steps leading to a valuation of the nearly 130,000 congregation-based recovery support groups for people struggling with drug or alcohol abuse using data from A.A. as a proxy. The basic building block is the number of people who have been saved from death by these groups. We know that A.A. reports 1,297,396 members in the USA (Alcoholics Anonymous 2018b, May). If we were to count each one of these members as a life saved and then apply the same VSL used by the CEA (2017) (i.e., low, $5.4 million; middle, $9.6 million; and high, $13.4 million) to estimate the cost associated with overdose mortality, this would equal a low estimate of $7.0 trillion, a middle of $12.5 trillion, and a high of $17.4 trillion. These figures are of course unreasonable valuations for several reasons. First, the high estimate is nearly equal to the entire US economy. Second, not all of these people would have died due to substance abuse. Finally, they do not represent the actual number of people in congregational programs, which likely equals or exceeds the A.A. membership figure.

We now offer a more reasonable way of estimating the lives saved and the statistical value of those lives through a series of steps using the data summarized in Table 2 (see Appendix Table 6, for calculations). First, we begin by breaking down the A.A. *total membership* by *age structure*, knowing that people die at different rates according to age. We also know that people addicted to alcohol are much more likely to die than those who are not. We then apply the *relative mortality rate* to people with alcohol use disorder, which is estimated to be 3.45 higher than that of sober people (Laramée et al. 2015), and arrive at the excess deaths for each age-group, which would have occurred had it not been for A.A. However, to assume that all people in A.A. will stay sober and reduce their risk of death is unreasonable, given that some A.A. members relapse and thus put themselves at a higher risk. Adopting a conservative approach, we take into our calculation only A.A. members who have been sober for 5 years or more and are likely to stay sober. According to Dennis et al. (2007), 86% of people reaching this threshold tend to remain sober.

To explain our calculations, we will first discuss the process without taking age differences into account. In the overall US population, 849.3 people per 100,000 die yearly according to the latest mortality data (Xu et al. 2018). This means that out of the total A.A. membership of 1,297,396 people (Alcoholics Anonymous 2018b, May), 11,019 can be expected to die due to all causes (e.g., age, accident, disease, etc.). However, if all these A.A. members were still addicted to alcohol (i.e., had alcohol use disorder), the mortality rate would be three to four times higher or, as estimated by Laramée et al. (2015), 3.45 times higher, which would be 38,015 people. This is 26,996 more deaths than would be generally expected; in other words, these are 26,996 people who would have possibly died but did not because they were the sober members of A.A. We could stop here; however, to be more conservative in our estimate, realizing that there are high rates of relapse in the first years of sobriety, we will focus on counting as successful only 49% of that total (13,228), which is the share of A.A. members who have achieved five or more years of sobriety. Moreover, even among those achieving 5 years or more of sobriety, 86% are likely to relapse (Dennis et al. 2007). Applying this additional condition means that 11,376 people are alive this year who otherwise would not have been without achieving sobriety.

Using a similar process, we now incorporate into the calculations A.A. membership age differences from the 2016 A.A. membership survey. Combining these data with age- and gender-specific mortality data (Xu et al. 2018) will yield an age-adjusted total estimate of 9878 people alive this year who otherwise would not have been without achieving sobriety through A.A. (see Appendix Table 6, for calculations). This estimation may seem by some as overly conservative, especially because a common story of A.A. members is that were it not for A.A., they would be in jail, institutionalized, or dead. Nevertheless, it is appropriate to incorporate into our estimates these theoretically and empirically relevant factors.

The age-adjusted estimate of 9878 lives saved through A.A. annually provides a proxy that can be used to estimate the economic impact of congregation-based recovery groups. Dividing this number by the 61,904 A.A. groups in the USA (Alcoholics Anonymous 2018b, May) indicates that 0.16 lives are saved per group each year. Multiplying this figure of 0.16 lives per group by the 129,680 faith congregations with recovery groups provides an estimate of 20,693 lives saved each year. Taking this figure and applying the VSL from the CEA provide three age-adjusted estimates of the value of these congregational efforts: low, $111.7 billion; middle, $198.6 billion; and high, $277.3 billion (see Table 3).

**Table 3:** Estimated annual valuation of congregational substance abuse recovery programs. *Sources*: A.A. (2014, 2018, May), CEA (2017), Dennis et al. (2007), Grim and Grim (2016), Laramée et al. (2015) and National Vital Statistics Reports (Xu et al. 2018). *CEA Sources*: Aldy and Viscusi (2008), US DOT (2016a, b), CDC WONDER database, multiple cause of death files, Substance Abuse and Mental Health Services Administration (2016) and Ruhm (2017)

VSL assumption	Fatalities prevented ($ in billion)	Non-fatality value ($ in billion)	Total value ($ in billion)
Low	111.7	39.3	151.0
Middle	198.6	39.3	237.9
High	277.3	39.3	316.6

Fatalities prevented assumes 20,693 lives saved annually (0.16 lives per group in 129,680 faith congregations). This is then multiplied by the VSL used by the CEA (2017) (i.e., low, $5.4 million; middle, $9.6 million; and high, $13.4 million). Non-fatality value assumes 10.1 persons per group stay sober in a given year across the 129,680 congregational support groups, equaling 1,309,463 people. Using the CEA’s estimate for non-fatality costs ($30,000 each), this equals $39.3 billion

Further, following the CEA’s estimate of non-fatality costs, we can also consider the shorter-term fatality prevention benefit of those who have been sober. Dennis et al. (2007) found that 66% of the alcoholics who remain sober for 1 year or more did not relapse. We can use this as a reasonable estimate of the number of people who remain sober in any given year. The 2016 A.A. survey reports that 73% of A.A. members are sober for 1 year or more, which equals 947,099 people. If 66% of these do not relapse during the year, we can estimate that 625,085 people are kept from entering the rehab or criminal justice systems. Turning that into a per-group number would be 10.1 persons per group; across the 129,680 congregational support groups, that would be 1,309,463 people. Using the CEA’s estimate for *non*-*fatality costs of addiction* ($30,000 each), this would be $39.3 billion worth of value (see Table 3). Adding this to the VSL estimates yields the total annual valuations of congregational recovery support groups at a low $151.0 billion, a middle $237.9 billion, and a high $316.6 billion.

Volunteer addiction recovery support groups meeting in congregations around the USA contribute up to $316.6 billion in benefit to the US economy every year *at no cost to tax payers.* And this represents only a portion of the faith-based work addressing the addiction crisis.

### Discussion

This study shows that religious beliefs, practices, and ministries not only provide succor and solace to those in need; they provide tangible, valuable resources that can help prevent and address substance abuse. This study also shows that the estimate by Grim and Grim (2016) of the value of religion’s individual impact ($158.8 billion), which includes everything from marital counseling to employment services to addiction recovery support, is too low. Specifically, this study’s middle estimate of $237.9 billion for substance abuse programs *alone* is 33% higher than their total individual social impact category. This therefore confirms that Johnson’s (2016) argument for a higher valuation, mentioned above, is correct.

**Fig. 4:**
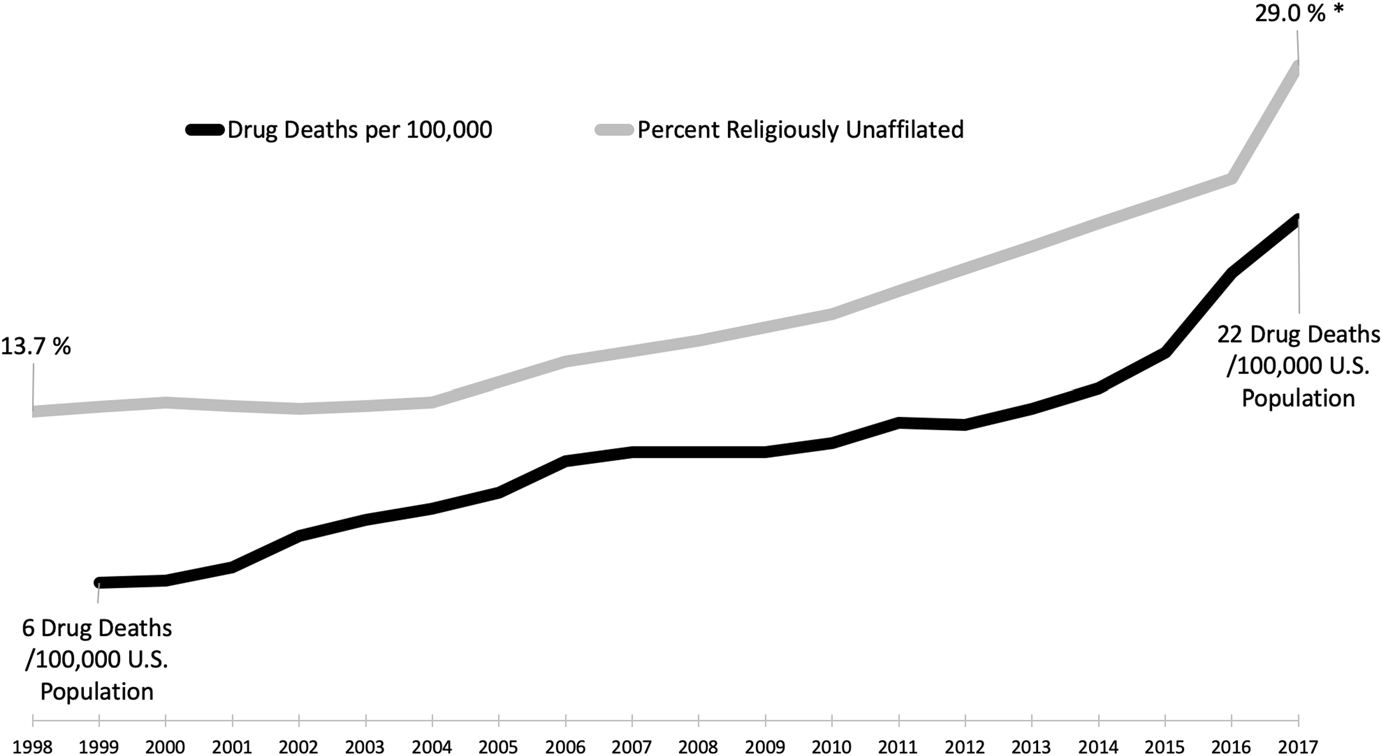
Drug deaths per 100,000 versus percentage of US Population religiously unaffiliated. *Dug death data*: National Institute on Drug Abuse (2018); 2017 data provisional. *Religiously unaffiliated data*: General Social Survey 1998–2012; PRRI 2013–2016; Pew Research Center (2018). *Data for 2017 are for *nonreligious,* i.e., people who hold virtually no religious belief and/or who view religion negatively (Pew 2018), which is a slightly different category than *religiously unaffiliated* used for years 1998–2016. We include it because it is consistent with the general trend toward religious disaffiliation.

When considering the role of religion in substance abuse prevention and recovery, it is impossible not to reflect on a provocative correlation: Americans are simultaneously identifying with religion less and suffering from substance abuse more (see Fig. 4). Over the past decade, the proportion of the US population that is unaffiliated with any religious group has risen sharply from 13.7% in 1998 to 24% in 2016, with the Pew Research Center (2018) estimating that 29% of American adults in 2017 were non-religious (see Fig. 4).^8^ The growth of the religiously unaffiliated population is occurring across multiple demographic groups; however, it is more concentrated among millennials (those born between 1981 and 1996), 35% of whom identify themselves as religiously unaffiliated (Pew Research Center 2015). This is particularly worrying because about one out of every six American young adults (aged between 18 and 25) battled a substance use disorder in 2014, which represents the highest percentage out of any age-group (Thomas 2018). Over the same time period, as shown in Fig. 4, drug overdose deaths rose from 6 per 100,000 in 1999 to 20 deaths per 100,000 in 2016, and the National Institute on Drug Abuse (2018) reports an estimated 72,306 overdose deaths in the USA in 2017, which is 22 deaths per 100,000.

These data and the evidence this study presents indicate that the decline in religious affiliation in the USA is a national health concern and not merely a concern for the religious groups.

Coinciding with this decreased religious involvement and increased substance abuse mortality is a significant drop from 67% in 1998 to 46% in 2018 of the Americans who think that religion can answer today’s problems (Brenan 2018). This marks the first time in more than six decades of polling that Gallup found that fewer than half of Americans believe that religion can answer all or most of today’s problems. At the same time, the poll found a significant rise (from 20% in 1998 to 39% in 2018) in the belief among Americans that religion is largely out of date.

This study directly challenges current public perceptions on the efficacy of religion to solve today’s problems, which appear to be driven by a lack of familiarity with religion. Indeed, significant differences in opinion exist depending on how religiously active a person is, with 81% of those attending church every week reporting that religion can answer today’s problems compared with only 27% of people who attend church less often than once per month and 9% of those who have no religious affiliation, according to the Gallup poll.

Based on the effectiveness of faith-based initiatives to address substance abuse, it is justifiable that public spending supports their work. Indeed, the benefits of publicly supported faith-based social interventions are well documented (Hein 2014). Faith-based organizations are eligible to compete for government grants on the same basis as all other non-governmental organizations. However, faith-based organizations may use government grants only to the extent that the money goes to non-religious activities in furtherance of a predetermined social service. The delineation between inherently religious activities and government-funded services can be established where both operate at distinct times and venues. Of course, given that religion is part of the solution to the addiction crisis, this dividing wall may come at the cost of lower effectiveness when the power of religious beliefs and practices is segregated from treatment.

Currently, the federal government does not provide data that differentiate between faith-based and non-faith-based grantees. However, a decade ago, the White House (White House Faith-Based and Community Initiatives 2007) collected data on competitive non-formula grant awards in 138 federally administered programs and identified 35 program areas at eleven federal agencies, including the US Department of Housing and Urban Development (HUD), Health and Human Services (HHS), Education (ED), Justice (DOJ), Labor (DOL), Agriculture (USDA), Commerce, Agency for International Development (USAID), Small Business Administration (SBA), Veterans Affairs (VA), and the Corporation for National and Community Service. As shown in Table 4, nearly 11% of federal grant funds went to faith-based organizations (2007). The proportion today is likely in the same ballpark, indicating that much of the work of faith-based organizations to address substance abuse is accomplished with relatively little government funding.

**Table 4:** Federal competitive funding won by faith-based and secular nonprofit organizations (NPOs). FY07 (review of 138 competitive programs). *Source*: White House Faith-Based and Community Initiatives (2007)

Agency	Total awarded ($)	Faith-based NPOs ($)	(%)	Secular NPOs ($)	(%)	Other* ($)	(%)
HHS	10,362,789,431	817,684,162	7.9	6,972,494,505	67.3	2,572,610,764	24.8
USAID	4,333,719,700	585,281,010	13.5	3,405,029,352	78.6	343,409,338	7.9
HUD	2,129,128,572	513,223,573	24.1	1,291,632,588	60.7	324,272,411	15.2
USDA	1,544,586,548	83,756,451	5.4	442,658,533	28.7	1,018,171,564	65.9
DOJ	633,509,595	70,632,916	11.1	328,624,911	51.9	234,251,768	37.0
DOL	250,275,599	23,817,232	9.5	163,423,221	65.3	63,035,146	25.2
ED	190,246,245	11,712,236	6.2	72,771,756	38.3	105,762,253	55.5
CNCS	538,007,871	62,739,528	11.7	320,237,710	59.5	155,030,633	28.8
DOC	291,113,568	4,952,000	1.7	51,173,206	17.6	234,988,362	80.7
VA	88,970,254	33,655,168	37.8	49,846,764	56.0	$5,468,322	6.2
SBA	12,338,998	656,900	5.3	10,639,180	86.2	$1,042,918	8.5
TOTAL	20,374,686,382	2,208,111,177	10.8	13,108,531,727	64.3	5,058,043,478	24.9

*Other institutions include educational institutions, state and local governments, and others

## Conclusions

Lifesaving medicines and psychological interventions are important parts of rescue and recovery; however, they are not enough. Religion and religious participation can address the many issues that lead people to alcohol and/or drug dependency that medical interventions alone can fail to address. The evidence we have reviewed and presented above shows that religious beliefs, practices, and belonging as well as spiritual programs inspired by faith in a Higher Being significantly contribute to the prevention of and recovery from substance abuse. This study finds that 73% of substance abuse recovery programs in the USA include a spirituality-based element, as embodied in the 12-step programs and fellowships, the majority of which emphasize reliance on God or a Higher Power to stay sober. Addicts with a faith or spirituality heal faster.

In addition to the efficacious role of spirituality, congregations and faith-based institutions are particularly effective in community mobilization and timely response to crises. Faith communities are adept at facilitating quality group interactions focused on overcoming past negative experiences, which are often drivers of the emotional and spiritual despondency that feed mental illness and substance abuse. This study found that volunteer addiction recovery groups meeting in congregations across the USA contribute up to $316.6 billion in savings to the US economy every year *at no cost to tax payers.* Based on the effectiveness of faith-based initiatives to address substance abuse, it is justifiable that public spending support their work.

Although negative experiences with religion (e.g., clergy sex abuse and other horrendous examples) have been a contributory factor to substance abuse among some victims, given that more than 84% of scientific studies show that faith is a positive factor in addiction prevention or recovery and a risk in less than 2% of the studies reviewed, we conclude that religion and spirituality are exceptionally powerful, integral, and indispensable resources in substance abuse prevention and recovery; faith plays a key role in treating the mind, body, and spirit. Therefore, the decline in religious affiliation in the USA is a national health concern.

## Appendix

See Tables 5 and 6.

**Table 5:** A.A. Meetings held in properties provided by congregations in Annapolis, MD *Source*: Annapolis Area Intergroup (2017, August)

Congregation	Meetings/week	Congregation	Meetings/week
All Hallows Church	1	Nichols Bethel Church	1
All Saints Episcopal Church	2	Old Wye Episcopal Church	1
Ark and Dove Presbyterian Church	1	Our Lady of Chesapeake Catholic Church	1
Arnold Asbury Methodist Church	3	Our Shepherd Lutheran Church	1
Arundel House of Hope	1	Presbyterian Church	2
Asbury United Methodist Church	1	Prince of Peace Presbyterian Church	1
Bethel Methodist Church	1	Sacred Heart Church	1
Broadneck Baptist Church	1	Samaritan House	1
Brooklyn Heights Methodist Church	1	Sarah’s House	1
Calvary United Methodist Church	4	Severna Park Baptist Church	1
Cavalry Chapel	1	Severna Park United Methodist Church	3
Celebrate Recovery Bookstore	9	St. Alban’s Church	2
Centenary United Methodist Church	1	St. Andrew’s Episcopal Church	1
Christ Church	1	St. Andrews Church	3
Christ Lutheran Church	2	St. Ann’s Parish Hall	1
Church of the Crucifixion	1	St. Bernadette Parish	6
College Parkway Baptist Church	4	St. Christopher’s Episcopal Church	1
Community United Methodist Church	6	St. Francis de Chantel Catholic Church	1
Davidsonville United Methodist Church	1	St. James Episcopal Church	2
Eastport Methodist Church	1	St. John’s Lutheran Church	2
Emanuel Lutheran Church	1	St. Laurence Martyr Parish Center	1
First Evangelist Lutheran Church	1	St. Luke’s Church	2
First Presbyterian Church and its Red House	20	St. Margaret’s Church	1
Fowler United Methodist Church	1	St. Martins in the Field	2
Galesville United Methodist Church	1	St. Martins Lutheran Church	2
Glen Lutheran Church	1	St. Mary’s Catholic Church	3
Heritage Baptist Church	2	St. Paul’s Lutheran Church	6
Holy Family Catholic Family	1	St. Paul’s Methodist Church	1
Hope Presbyterian Church	1	St. Phillips Episcopal Church	5
Huntington Methodist Church	1	The Church of Jesus Christ of Latter-day Saints*	1
Kent Island United Methodist Church	1	The Salvation Army	2
Korean Presbyterian Church	1	Trinity Methodist Church	2
Linthicum Heights Methodist Church	1	Trinity Episcopal Church	1
Living Water Lutheran Church	1	Union Church	2
Magothy Methodist Church	1	Unitarian Universalist Church of Annapolis	1
Marley United Methodist Church	1	Unitarian Universalist Church of Annapolis - Wellbriety**	1
Mayo United Methodist Church	4	Village Baptist Church	2
Mt. Harmony United Methodist Church	2	Wesley Grove United Methodist Church	1
Mt. Olive Methodist Church	1	Woods Memorial Church	5

*Adapted version of A.A. 12 steps; **Native American A.A.-type meeting

**Table 6:** Estimate of lives saved (excess deaths avoided) through A.A. *Sources*: Length of sobriety (Alcoholic Anonymous 2014), risk of relapse over time (Dennis et al. 2007), age structure of A.A. members (Alcoholic Anonymous 2014), mortality rates (National Vital Statistics Reports, Xu et al. 2018), relative mortality risk of people with alcohol use disorder (Laramée et al. 2015), total membership (Alcoholics Anonymous 2018a), number of groups (Alcoholics Anonymous 2018a), VSL (Council of Economic Advisers 2017), non-fatality costs of addiction (Council of Economic Advisers 2017), number of religious and spiritual substance abuse recovery programs held in congregations (Grim and Grim 2016). *CEA Sources*: Aldy and Viscusi (2008), U.S. DOT (2016a, b), CDC WONDER database, multiple cause of death files, Substance Abuse and Mental Health Services Administration (2016) and Ruhm (2017)

		A.A. members	Normal deaths per 100K	Total likely deaths among 1.297 million people in general	Total likely deaths among 1.297 million people with alcohol use disorder	Excess deaths	Excess deaths among those sober five or more years	Excess deaths among those sober five or more years assuming 86% do not relapse
Not adjusted for age →		1,297,396	849.3	11,019	38,015	26,996	13,228	11,376
A.A. age structure								
Age ranges	% Members							
Under 21*	1	12,974	74.9	10	34	24	12	10
21–30	11	142,714	102.0	145	502	356	175	150
31–40	14	181,635	160.6	292	1006	715	350	301
41–50	21	272,453	298.9	814	2809	1995	977	841
51–60	28	363,271	644.7	2342	8079	5737	2811	2418
61–70	18	233,531	1336.2	3120	10,766	7645	3746	3222
70+	7	90,818	3131.7	2844	9812	6968	3414	2936
Age-adjusted total	100			9568	33,008	23,441	11,486	9878

## Footnotes

1 Grim and Grim (2016, p. 17) estimated that 129,680 faith congregations (which is 37.6% of an estimated total of 344,894 congregations) in the United States have groups for people struggling with drug or alcohol abuse.

2 Alcoholics Anonymous is an international fellowship of men and women who want to resolve their drinking problems. A.A.’s 12 steps encompass a group of principles, spiritual in nature, which A.A. advocates practicing as a way of life to dispel the obsession with alcohol and enable the sufferer to become happily and usefully whole (see https://www.aa.org/). Many different types of addiction recovery support programs have adopted and adapted the 12-step approach pioneered by A.A.

3 There is some debate whether A.A. is religious, although the group itself clearly states that it is not religious. However, the Second, Seventh, and Ninth United States courts have ruled that compelling inmates, parolees, and probationers to attend A.A. is unconstitutionally coercive and a violation of the Establishment Clause of the First Amendment of the US Constitution (United States Ninth Circuit 2007).

4 VSL is sometimes misleadingly referred to simply as “value of life.” This is erroneous because monetization does not actually place a “value” on individual lives because the value of any individual’s life cannot be expressed in monetary terms. The sole purpose is to help estimate the likely statistical benefits of a regulatory action that reduces the risks that people face.

5 The Office of Management and Budget (n.d., p. 10) advises US government agencies against the overly simplistic rationale for the monetization of health and safety benefits, such as the avoided cost of illness or avoided lost earnings. Instead, the measure should capture pain and suffering and other quality-of-life effects including, but not limited to, the private demand for prevention of the risk and the net financial externalities associated with the risk, such as net changes in public medical costs and any net changes in economic production that are not experienced by the target population.

6 The US DOT (2016b) uses a value of $9.6 million (according to the 2015 value of dollar) for each expected fatality reduction, with sensitivity analysis conducted at alternative values of $5.4 million and $13.4 million. The US Environmental Protection Agency’s (EPA) (2016) current guidance calls for using a VSL estimate of $10.1 million (according to the 2015 value of dollar). The US Department of Health and Human Services (HHS) (2016a, b) suggests using the range of estimates from Robinson and Hammitt (2016), a low of $4.4 million to a high of $14.3 million with a central value of $9.4 million (according to the 2015 value of dollar).

7 While A.A. does not provide data on the number of groups meeting in congregations, they do report on the over 66,000 A.A. groups in the USA and Canada, 61,904 of which are in the USA (Alcoholic Anonymous 2018a), around 1000 are in treatment facilities, and over 1400 are in correctional institutions (Alcoholic Anonymous 2018b, p. 3). The remainder is hosted in congregations, community centers, etc.

8 *Nonreligious* are people who hold virtually no religious belief and/or who view religion negatively (Pew 2018), which is a slightly different category than *religiously unaffiliated.*
